# Crystal structure of *N*,*N*′-bis­[(pyridin-4-yl)meth­yl]naphthalene di­imide

**DOI:** 10.1107/S1600536814017917

**Published:** 2014-08-09

**Authors:** Mariana Nicolas-Gomez, Diego Martínez-Otero, Alejandro Dorazco-González

**Affiliations:** a Centro Conjunto de Investigacion en Quimica Sustentable UAEM-UNAM, Instituto de Quimica, Universidad Nacional Autonoma de Mexico, Carretera Toluca-Atlacomulco Km 14.5 CP 50200 Toluca, Estado de Mexico, Mexico

**Keywords:** crystal structure, naphthalene di­imide, transistors, organic supra­molecular solids, hydrogen bonding, π–π stacking

## Abstract

In the centrosymmetric title compound, C_26_H_16_N_4_O_4_ {systematic name: 6,13-bis­[(pyridin-4-yl)meth­yl]-6,13-di­aza­tetra­cyclo­[6.6.2.0^4,16^0^11,15^]hexa­deca-1,3,8,10,15-pantaene-5,7,12,14-tetrone}, the central ring system is essentially planar [maximum deviation = 0.0234 (8) Å] and approximately perpendicular to the terminal pyridine ring [dihedral angle = 84.38 (3)°]. The mol­ecules displays a *trans* conformation with the (pyridin-4-yl)methyl groups on both sides of the central naphthalene di­imide plane. In the crystal, mol­ecules are linked by π–π stacking between parallel pyridine rings [centroid–centroid distances = 3.7014 (8) and 3.8553 (8) Å] and weak C—H⋯O hydrogen bonds, forming a three-dimensional supra­molecular architecture.

## Related literature   

For crystal structures of related compounds, see: Xu *et al.* (2011 ▶); Reczek *et al.* (2006 ▶); Li *et al.* (2009 ▶). For colorimetric applications and nanoscale properties, see: Pandeeswar *et al.* (2014 ▶); Trivedi *et al.* (2009 ▶); Matsunaga *et al.* (2014 ▶); Pantoş *et al.* (2007 ▶). For the design of transistors, see: Jung *et al.* (2009 ▶); Oh *et al.* (2010 ▶). For organic supra­molecular solids, see: Cheney *et al.* (2007 ▶). For the design and synthesis of one-dimensional coordination polymers, see: Li *et al.* (2011 ▶, 2012 ▶).
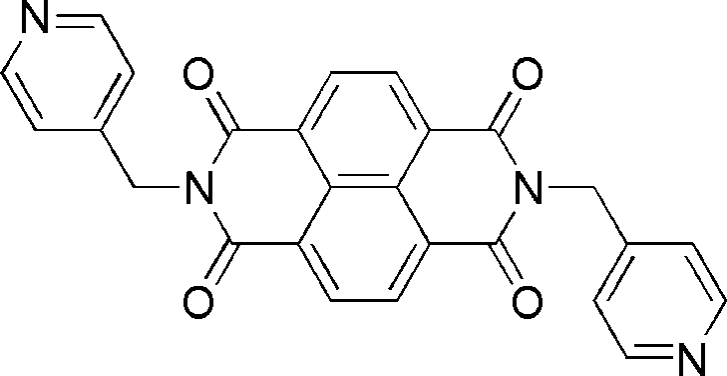


## Experimental   

### Crystal data  

C_26_H_16_N_4_O_4_
*M* *_r_* = 448.43
Triclinic, 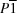

*a* = 5.5891 (4) Å
*b* = 7.5232 (5) Å
*c* = 11.9525 (8) Å
α = 77.093 (3)°
β = 88.445 (4)°
γ = 87.590 (4)°
*V* = 489.37 (6) Å^3^
*Z* = 1
Cu *K*α radiation
μ = 0.87 mm^−1^
*T* = 296 K
0.34 × 0.13 × 0.08 mm

### Data collection  

Bruker APEXII CCD diffractometer
Absorption correction: multi-scan (*SADABS*; Bruker, 2008 ▶) *T*_min_ = 0.555, *T*_max_ = 0.753
14032 measured reflections
1748 independent reflections
1643 reflections with *I* > 2σ(*I*)
*R*_int_ = 0.045

### Refinement  

*R*[*F* ^2^ > 2σ(*F* ^2^)] = 0.037
*wR*(*F* ^2^) = 0.106
*S* = 1.05
1748 reflections
154 parameters
H-atom parameters constrained
Δρ_max_ = 0.19 e Å^−3^
Δρ_min_ = −0.19 e Å^−3^

### 

Data collection: *APEX2* (Bruker, 2008 ▶); cell refinement: *SAINT* (Bruker, 2008 ▶); data reduction: *SAINT*; program(s) used to solve structure: *SHELXTL* (Sheldrick, 2008 ▶); program(s) used to refine structure: *SHELXTL*; molecular graphics: *SHELXTL*; software used to prepare material for publication: *SHELXTL*.

## Supplementary Material

Crystal structure: contains datablock(s) I, global. DOI: 10.1107/S1600536814017917/xu5806sup1.cif

Structure factors: contains datablock(s) I. DOI: 10.1107/S1600536814017917/xu5806Isup2.hkl

**Fig. 1.**
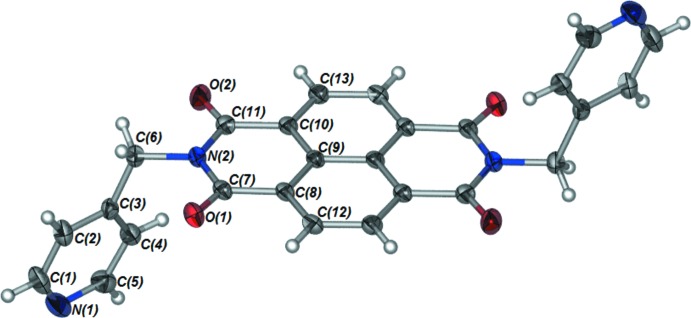
The structure with displacement ellipsoids drawn at the 30% probability level and H atoms as small sphere of arbitrary radii.

**Fig. 2.**
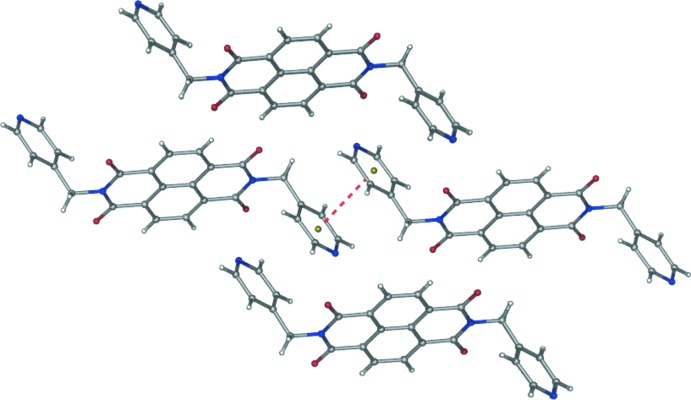
View p-stacking interactions in the crystal.

. DOI: 10.1107/S1600536814017917/xu5806fig1.tif
The structure with displacement ellipsoids drawn at the 30% probability level and H atoms as small sphere of arbitrary radii.

p . DOI: 10.1107/S1600536814017917/xu5806fig2.tif
View *p*-stacking inter­actions in the crystal.

CCDC reference: 1017799

Additional supporting information:  crystallographic information; 3D view; checkCIF report

## Figures and Tables

**Table 1:** Hydrogen-bond geometry (Å, °)

*D*—H⋯*A*	*D*—H	H⋯*A*	*D*⋯*A*	*D*—H⋯*A*
C4—H4⋯O1^i^	0.93	2.59	3.5014 (15)	165
C13—H13⋯O2^ii^	0.93	2.51	3.3242 (15)	146

Symmetry codes: (i) 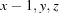; (ii) 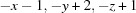.

## supplementary crystallographic information

### S1. Comment

The assembly of organic molecules in solid state has attracted much attention in supramolecular chemistry, especially in crystal engineering and materials science (Cheney *et al.*, 2007). The study and understanding of intermolecular interactions in a crystal is a central topic for design and synthesis of functional organic materials with desirable optical, electronic and structural properties (Pandeeswar *et al.*, 2014). Herein we report the crystal structure of the title compound, which was prepared from 1,4,5,8-naphthalene dianhydride and 4-aminomethyl-pyridine under reflux in dry DMF. Suitable single-crystals for X-ray diffraction, were obtained directly from reaction mixture after of 12 h. The compound with methanol solvate (1:1) has been reported (Li *et al.*, 2009) and was obtained as by-product during preparation of transition polymeric complexes in a mixture (methanol:chloroform) under solvothermal conditions, structurally this solvated is similar to compound reported here, both *trans* conformation of *N*-(4-pyridilmethyl) groups as to the dihedral angles between central ring and pyridil groups (86.34°). The title compound has also been studied as a semi-rigid ditopic ligand to the synthesis of one-dimensional coordination polymers with Zn(II), Mn(II), Co(II), Cd(II) where the ligand plays the key role to determine the final conformation of the polymeric structures (Li *et al.*, 2011, 2012). The coordination of (I) to the salts of ZnCl2, Zn(ClO_4_)_2_, Zn(CF_3_SO_3_)_2_ generates one-dimensional polymeric structures where *trans* conformation of (I) is maintained and not significant structural changes observed. A series of one-dimensional metal-organic frameworks of Mn(SCN)_2_, Co(SCN)_2_ and Cd(SCN)_2_ with (I) have been reported, in all cases (I) shows a *Z* mode conformation and links up two metal centers such that one-dimensional chains are formed with π-π stacking interactions (centroid-centroid distances = 3.92–4.14 Å).

### S2. Experimental

All chemicals were acquired commercially and were used without further purification. A mixture of 1,4,5,8-naphthalene dianhydride (1.0 g, 0.005 mol) and 4-aminomethyl-pyridine (1.08 g, 0.01 mol) in dry DMF (35 ml) was heated under reflux in atmosphere of dinitrogen and stirring for 2 h. Afterwards the resulting yellow solution was cooling and pallid yellow crystals were obtained on the wall of the flask directly from the mixture which corresponds to the compound I pure according to 1H NMR in DMSO-d6. Yield: 95%. Elemental analysis calculated (%) for C_26_H_16_N_4_O_4_; C, 69.64; H, 3.60; N, 12.49; found: C, 69.60; H, 3.62; N, 12.45.

### S3. Refinement

H atoms were placed in calculated positions with C—H = 0.93–0.97 Å, and refined in riding mode with U_iso_(H) = 1.2U_eq_(C).

### Crystal data

**Table:**

C_26_H_16_N_4_O_4_	*Z* = 1
*M**_r_* = 448.43	*F*(000) = 232
Triclinic, *P*1	*D*_x_ = 1.522 Mg m^−^^3^
*a* = 5.5891 (4) Å	Cu *K*α radiation, λ = 1.54178 Å
*b* = 7.5232 (5) Å	Cell parameters from 9981 reflections
*c* = 11.9525 (8) Å	θ = 3.8–68.3°
α = 77.093 (3)°	µ = 0.87 mm^−^^1^
β = 88.445 (4)°	*T* = 296 K
γ = 87.590 (4)°	Plate, colourless
*V* = 489.37 (6) Å^3^	0.34 × 0.13 × 0.08 mm

### Data collection

**Table:**

Bruker APEXII CCD diffractometer	1748 independent reflections
Radiation source: Incoatec ImuS	1643 reflections with *I* > 2σ(*I*)
Mirrors monochromator	*R*_int_ = 0.045
ω scans	θ_max_ = 68.3°, θ_min_ = 3.8°
Absorption correction: multi-scan (*SADABS*; Bruker, 2008)	*h* = −5→6
*T*_min_ = 0.555, *T*_max_ = 0.753	*k* = −9→9
14032 measured reflections	*l* = −14→14

### Refinement

**Table:**

Refinement on *F*^2^	0 restraints
Least-squares matrix: full	Hydrogen site location: inferred from neighbouring sites
*R*[*F*^2^ > 2σ(*F*^2^)] = 0.037	H-atom parameters constrained
*wR*(*F*^2^) = 0.106	*w* = 1/[σ^2^(*F*_o_^2^) + (0.0617*P*)^2^ + 0.0799*P*] where *P* = (*F*_o_^2^ + 2*F*_c_^2^)/3
*S* = 1.05	(Δ/σ)_max_ < 0.001
1748 reflections	Δρ_max_ = 0.19 e Å^−^^3^
154 parameters	Δρ_min_ = −0.19 e Å^−^^3^

### Special details

**Table:**

Geometry. All e.s.d.'s (except the e.s.d. in the dihedral angle between two l.s. planes) are estimated using the full covariance matrix. The cell e.s.d.'s are taken into account individually in the estimation of e.s.d.'s in distances, angles and torsion angles; correlations between e.s.d.'s in cell parameters are only used when they are defined by crystal symmetry. An approximate (isotropic) treatment of cell e.s.d.'s is used for estimating e.s.d.'s involving l.s. planes.

### Fractional atomic coordinates and isotropic or equivalent isotropic displacement parameters (Å^2^)

**Table:**

	*x*	*y*	*z*	*U*_iso_*/*U*_eq_	
O1	0.43898 (16)	0.61174 (13)	0.75255 (7)	0.0471 (3)	
N1	−0.0333 (2)	0.68325 (18)	1.12186 (9)	0.0587 (3)	
C1	0.1698 (3)	0.7665 (2)	1.09030 (12)	0.0600 (4)	
H1	0.2727	0.7785	1.1478	0.072*	
O2	−0.21818 (18)	0.96340 (12)	0.62202 (8)	0.0540 (3)	
N2	0.10767 (18)	0.78494 (13)	0.68880 (8)	0.0375 (3)	
C2	0.2394 (2)	0.83681 (19)	0.97785 (11)	0.0482 (3)	
H2	0.3847	0.8938	0.9613	0.058*	
C6	0.1602 (2)	0.90343 (17)	0.76740 (10)	0.0425 (3)	
H6A	0.0742	1.0198	0.7428	0.051*	
H6B	0.3301	0.9262	0.7628	0.051*	
C3	0.0913 (2)	0.82151 (15)	0.89059 (9)	0.0372 (3)	
C5	−0.1744 (3)	0.66899 (19)	1.03678 (12)	0.0511 (3)	
H5	−0.3183	0.6110	1.0560	0.061*	
C4	−0.1216 (2)	0.73457 (17)	0.92155 (10)	0.0435 (3)	
H4	−0.2276	0.7204	0.8658	0.052*	
C7	0.2667 (2)	0.63581 (16)	0.69075 (9)	0.0360 (3)	
C8	0.2169 (2)	0.51409 (15)	0.61294 (9)	0.0337 (3)	
C9	0.02134 (19)	0.55699 (14)	0.53820 (8)	0.0315 (3)	
C10	−0.1324 (2)	0.71073 (15)	0.53795 (9)	0.0340 (3)	
C11	−0.0899 (2)	0.83074 (15)	0.61813 (9)	0.0376 (3)	
C12	0.3627 (2)	0.36316 (16)	0.61186 (9)	0.0383 (3)	
H12	0.4906	0.3354	0.6618	0.046*	
C13	−0.3204 (2)	0.74975 (16)	0.46403 (10)	0.0384 (3)	
H13	−0.4206	0.8516	0.4641	0.046*	

### Atomic displacement parameters (Å^2^)

**Table:**

	*U*^11^	*U*^22^	*U*^33^	*U*^12^	*U*^13^	*U*^23^
O1	0.0449 (5)	0.0592 (6)	0.0403 (5)	−0.0028 (4)	−0.0085 (4)	−0.0169 (4)
N1	0.0686 (8)	0.0691 (8)	0.0368 (6)	0.0111 (6)	0.0030 (5)	−0.0118 (5)
C1	0.0676 (10)	0.0771 (10)	0.0401 (7)	0.0099 (7)	−0.0136 (6)	−0.0243 (7)
O2	0.0626 (6)	0.0463 (5)	0.0580 (6)	0.0133 (4)	−0.0097 (4)	−0.0237 (4)
N2	0.0465 (6)	0.0387 (5)	0.0292 (5)	−0.0039 (4)	0.0003 (4)	−0.0113 (4)
C2	0.0479 (8)	0.0572 (8)	0.0454 (7)	0.0002 (5)	−0.0060 (5)	−0.0234 (6)
C6	0.0530 (7)	0.0405 (6)	0.0372 (6)	−0.0087 (5)	0.0007 (5)	−0.0143 (5)
C3	0.0436 (6)	0.0356 (6)	0.0358 (6)	0.0031 (4)	−0.0018 (4)	−0.0156 (4)
C5	0.0508 (8)	0.0541 (7)	0.0456 (7)	0.0033 (6)	0.0064 (6)	−0.0074 (6)
C4	0.0454 (7)	0.0467 (7)	0.0391 (6)	−0.0006 (5)	−0.0036 (5)	−0.0108 (5)
C7	0.0376 (6)	0.0437 (6)	0.0264 (5)	−0.0056 (4)	0.0017 (4)	−0.0069 (4)
C8	0.0368 (6)	0.0388 (6)	0.0249 (5)	−0.0027 (4)	0.0023 (4)	−0.0060 (4)
C9	0.0349 (6)	0.0351 (6)	0.0235 (5)	−0.0026 (4)	0.0032 (4)	−0.0044 (4)
C10	0.0384 (6)	0.0356 (6)	0.0272 (5)	−0.0012 (4)	0.0026 (4)	−0.0055 (4)
C11	0.0436 (7)	0.0364 (6)	0.0325 (6)	−0.0003 (5)	0.0021 (4)	−0.0077 (4)
C12	0.0376 (6)	0.0453 (6)	0.0304 (6)	0.0027 (5)	−0.0051 (4)	−0.0055 (5)
C13	0.0420 (6)	0.0376 (6)	0.0346 (6)	0.0061 (5)	−0.0002 (4)	−0.0071 (4)

### Geometric parameters (Å, º)

**Table:**

N1—C1	1.325 (2)	C5—H5	0.9300
N1—C5	1.3293 (19)	C6—H6A	0.9700
N2—C11	1.3931 (16)	C6—H6B	0.9700
N2—C7	1.3982 (16)	C7—C8	1.4823 (16)
N2—C6	1.4746 (14)	C8—C12	1.3718 (16)
O1—C7	1.2123 (14)	C8—C9	1.4110 (16)
O2—C11	1.2127 (15)	C9—C10	1.4113 (16)
C1—C2	1.382 (2)	C9—C9^i^	1.416 (2)
C1—H1	0.9300	C10—C13	1.3708 (17)
C2—C3	1.3766 (17)	C10—C11	1.4855 (16)
C2—H2	0.9300	C12—C13^i^	1.4046 (17)
C3—C4	1.3836 (18)	C12—H12	0.9300
C3—C6	1.5100 (16)	C13—C12^i^	1.4046 (17)
C4—C5	1.3834 (18)	C13—H13	0.9300
C4—H4	0.9300		
			
C1—N1—C5	115.61 (12)	C3—C6—H6B	109.0
C11—N2—C7	125.48 (10)	H6A—C6—H6B	107.8
C11—N2—C6	118.44 (10)	O1—C7—N2	120.35 (10)
C7—N2—C6	116.06 (10)	O1—C7—C8	122.58 (11)
N1—C1—C2	124.53 (13)	N2—C7—C8	117.06 (10)
N1—C1—H1	117.7	C12—C8—C9	120.29 (11)
C2—C1—H1	117.7	C12—C8—C7	120.26 (11)
C3—C2—C1	119.25 (13)	C9—C8—C7	119.44 (10)
C3—C2—H2	120.4	C8—C9—C10	121.50 (11)
C1—C2—H2	120.4	C8—C9—C9^i^	119.18 (13)
C2—C3—C4	117.23 (11)	C10—C9—C9^i^	119.32 (13)
C2—C3—C6	120.00 (11)	C13—C10—C9	120.29 (11)
C4—C3—C6	122.74 (11)	C13—C10—C11	119.95 (10)
C5—C4—C3	118.95 (12)	C9—C10—C11	119.77 (11)
C5—C4—H4	120.5	O2—C11—N2	120.98 (11)
C3—C4—H4	120.5	O2—C11—C10	122.31 (11)
N1—C5—C4	124.44 (13)	N2—C11—C10	116.70 (10)
N1—C5—H5	117.8	C8—C12—C13^i^	120.51 (11)
C4—C5—H5	117.8	C8—C12—H12	119.7
N2—C6—C3	112.85 (9)	C13^i^—C12—H12	119.7
N2—C6—H6A	109.0	C10—C13—C12^i^	120.41 (10)
C3—C6—H6A	109.0	C10—C13—H13	119.8
N2—C6—H6B	109.0	C12^i^—C13—H13	119.8
			
C5—N1—C1—C2	0.1 (2)	C12—C8—C9—C10	179.61 (9)
N1—C1—C2—C3	0.0 (2)	C7—C8—C9—C10	−1.78 (16)
C11—N2—C6—C3	104.43 (12)	C12—C8—C9—C9^i^	−0.36 (19)
C7—N2—C6—C3	−76.70 (13)	C7—C8—C9—C9^i^	178.25 (10)
C1—C2—C3—C4	−0.20 (18)	C8—C9—C10—C13	179.67 (9)
C1—C2—C3—C6	177.80 (12)	C9^i^—C9—C10—C13	−0.36 (19)
N2—C6—C3—C2	139.13 (11)	C8—C9—C10—C11	−0.25 (17)
N2—C6—C3—C4	−42.99 (16)	C9^i^—C9—C10—C11	179.72 (10)
C1—N1—C5—C4	−0.1 (2)	C7—N2—C11—O2	−179.77 (10)
N1—C5—C4—C3	0.0 (2)	C6—N2—C11—O2	−1.01 (17)
C2—C3—C4—C5	0.19 (17)	C7—N2—C11—C10	−0.43 (17)
C6—C3—C4—C5	−177.75 (11)	C6—N2—C11—C10	178.33 (9)
C11—N2—C7—O1	177.16 (10)	C13—C10—C11—O2	0.79 (18)
C6—N2—C7—O1	−1.63 (16)	C9—C10—C11—O2	−179.29 (10)
C11—N2—C7—C8	−1.55 (16)	C13—C10—C11—N2	−178.55 (9)
C6—N2—C7—C8	179.67 (8)	C9—C10—C11—N2	1.38 (16)
O1—C7—C8—C12	2.58 (17)	C9—C8—C12—C13^i^	0.44 (17)
N2—C7—C8—C12	−178.75 (9)	C7—C8—C12—C13^i^	−178.16 (9)
O1—C7—C8—C9	−176.03 (10)	C9—C10—C13—C12^i^	0.29 (18)
N2—C7—C8—C9	2.64 (15)	C11—C10—C13—C12^i^	−179.78 (10)

Symmetry code: (i) −*x*, −*y*+1, −*z*+1.

### Hydrogen-bond geometry (Å, º)

**Table:**

*D*—H···*A*	*D*—H	H···*A*	*D*···*A*	*D*—H···*A*
C4—H4···O1^ii^	0.93	2.59	3.5014 (15)	165
C13—H13···O2^iii^	0.93	2.51	3.3242 (15)	146

Symmetry codes: (ii) *x*−1, *y*, *z*; (iii) −*x*−1, −*y*+2, −*z*+1.
