# Supplementary figures and images for: Crystal structure of N,N′-bis­[(pyridin-4-yl)meth­yl]naphthalene di­imide
Source: Acta Crystallogr Sect E Struct Rep Online. 2014 Aug 9;70(Pt 9):o985–6. doi: 10.1107/S1600536814017917 (PMC4186118; doi:10.1107/S1600536814017917)

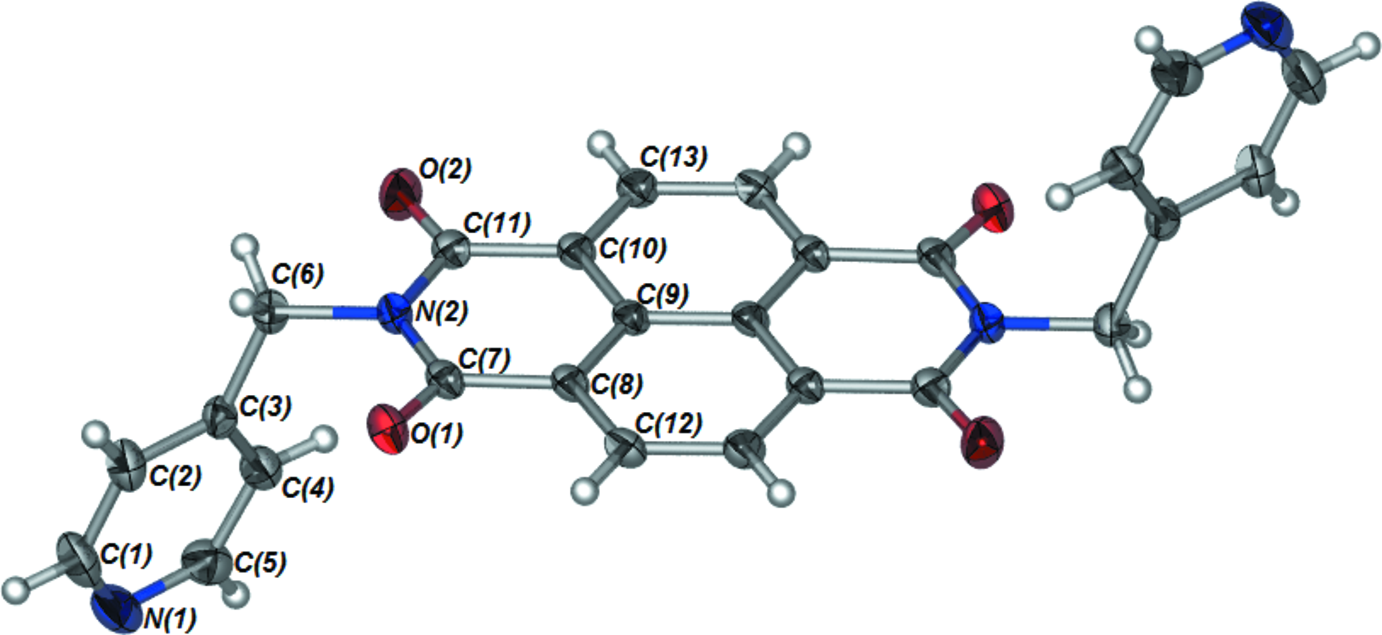

Supplement: Supplementary file 3 [file e-70-0o985-fig1.tif]

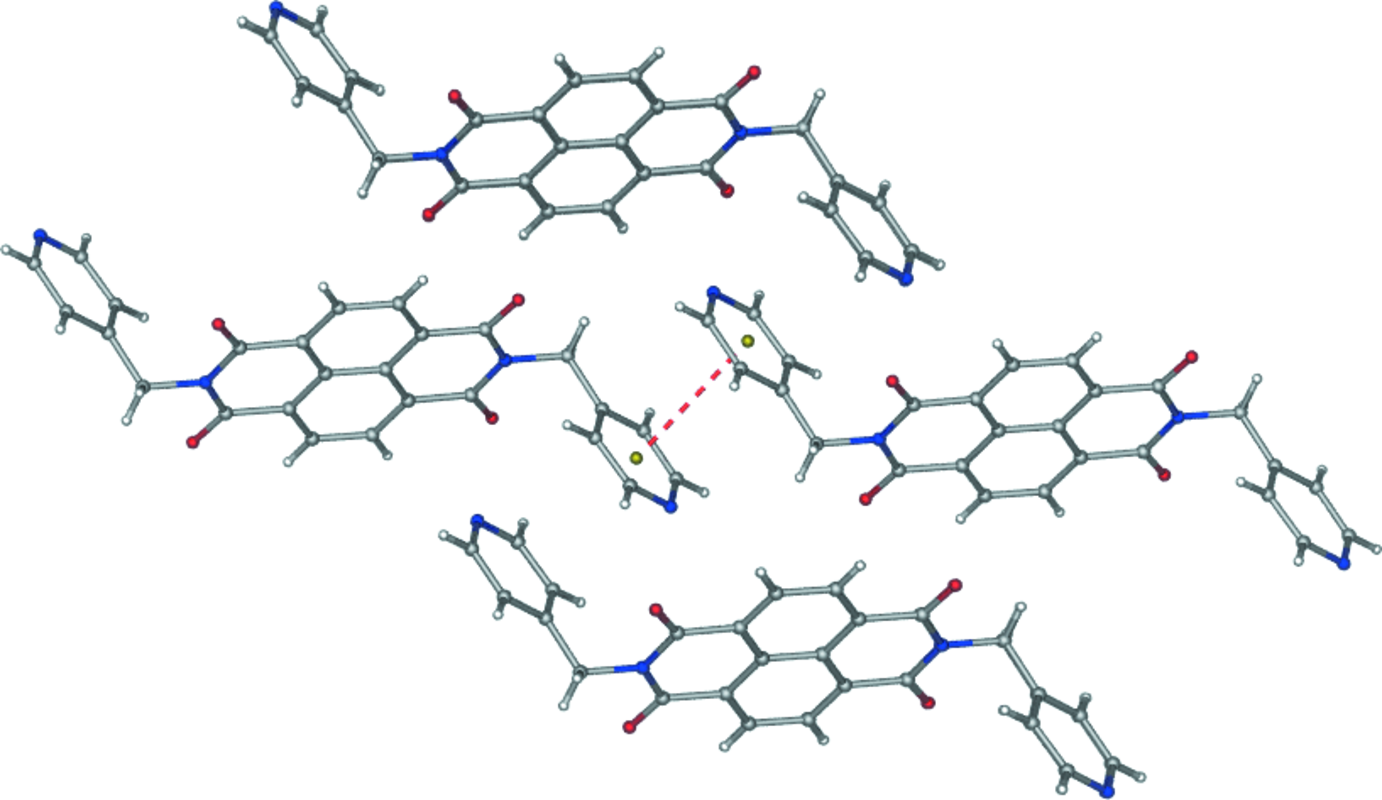

Supplement: Supplementary file 4 [file e-70-0o985-fig2.tif]
